# Supplementary material for: Design and characterisation of photoactivatable and lysine reactive o-nitrobenzyl alcohol-based crosslinkers
Source: Chem Sci. 2025 Jul 16;16(33):15239–55. doi: 10.1039/d5sc03211c (PMC12301895; doi:10.1039/d5sc03211c)
Supplement: SC-016-D5SC03211C-s001 [file SC-016-D5SC03211C-s001.pdf]

Supplementary Information (SI) for Chemical Science.
This journal is © The Royal Society of Chemistry 2025

Supporting Information

Design and Characterisation of Photoactivatable and Lysine Reactive *o*-Nitrobenzyl Alcohol-Based Crosslinkers

Adam Cahill[1], Martin Walko[2], Benjamin Fenton[2], Sri Ranjani Ganji[1], Anne Herbert[1], Sheena E. Radford[1], Nikil Kapur[3], Keith Livingstone[2], Megan H. Wright[2]*, Antonio N. Calabrese[1]*

[1] Astbury Centre for Structural Molecular Biology, School of Molecular and Cellular Biology, Faculty of Biological Sciences, University of Leeds, LS2 9JT, Leeds, United Kingdom

[2] Astbury Centre for Structural Molecular Biology, School of Chemistry, Faculty of Engineering and Physical Sciences, University of Leeds, LS2 9JT, Leeds, United Kingdom

[3] School of Mechanical Engineering, Faculty of Engineering and Physical Sciences, University of Leeds, Leeds LS2 9JT, U.K.

Correspondence to: Keith Livingstone (k.livingstone@leeds.ac.uk), Megan Wright (m.h.wright@leeds.ac.uk) and Antonio Calabrese (a.calabrese@leeds.ac.uk)

NMR Spectra of Synthesised Compounds

4-(hydroxymethyl)-3-nitro-N-2-propyn-1-ylbenzamide (1a)

^{1}H NMR (500 MHz, DMSO-d_6)

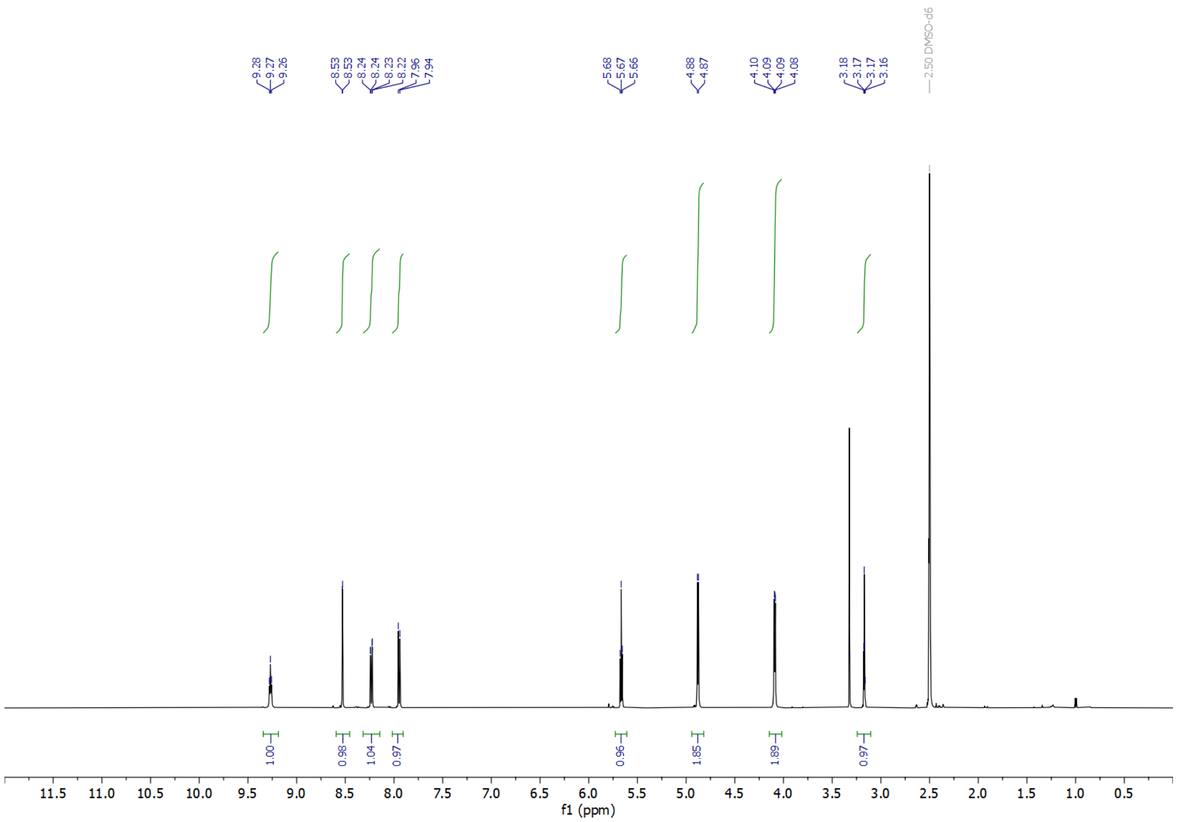

^{13}C NMR (101 MHz, DMSO-d_6)

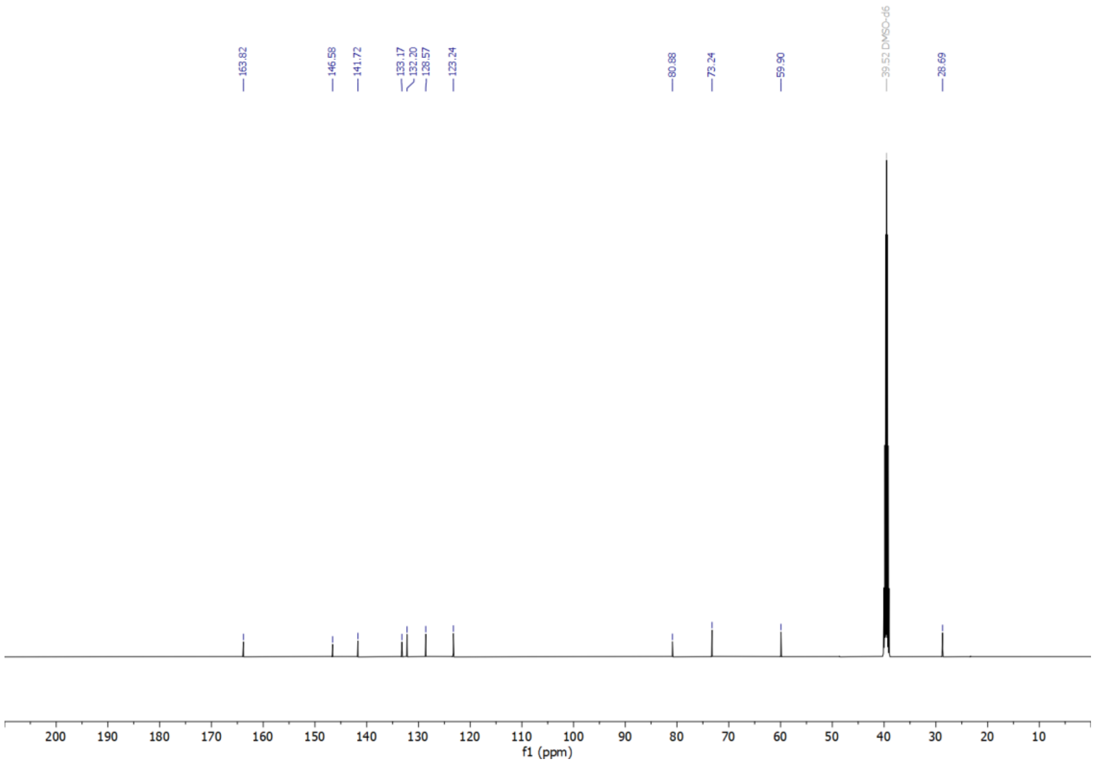

(2-Nitro-5-(prop-2-yn-1-yloxy)phenyl)methanol (1b)

^{1}H NMR (500 MHz, DMSO-d_6)

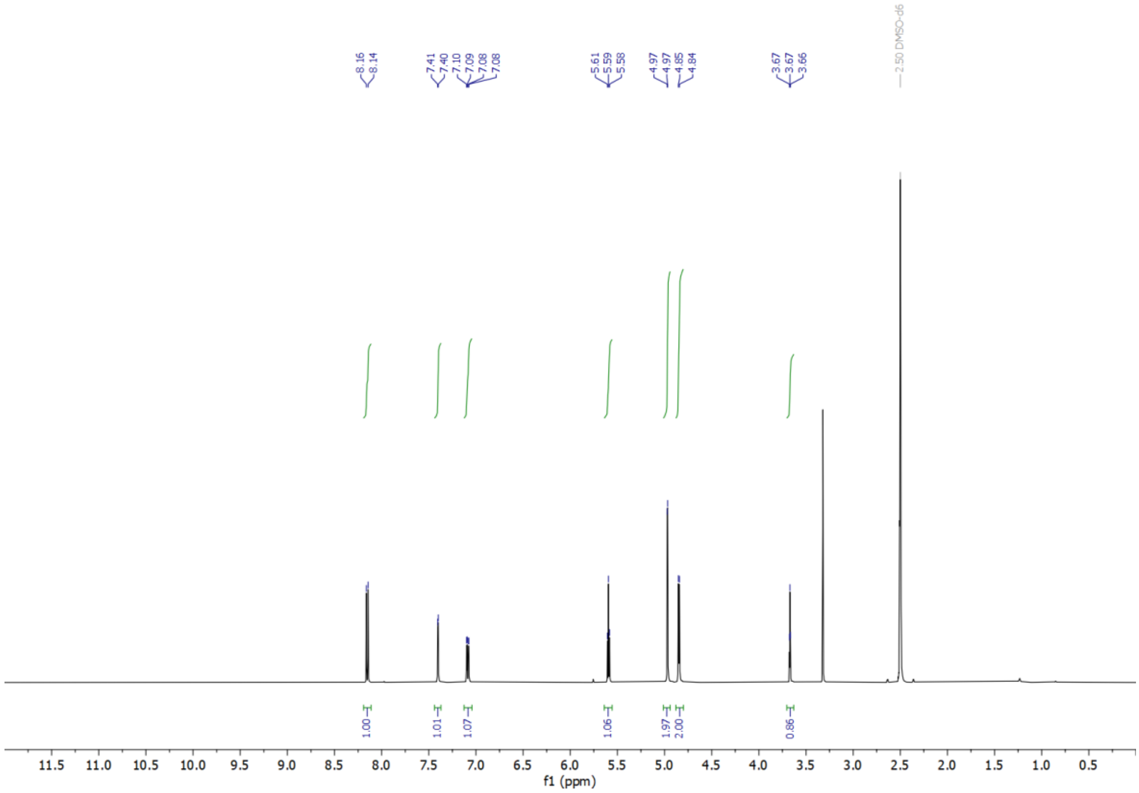

^{13}C NMR (101 MHz, DMSO-d_6)

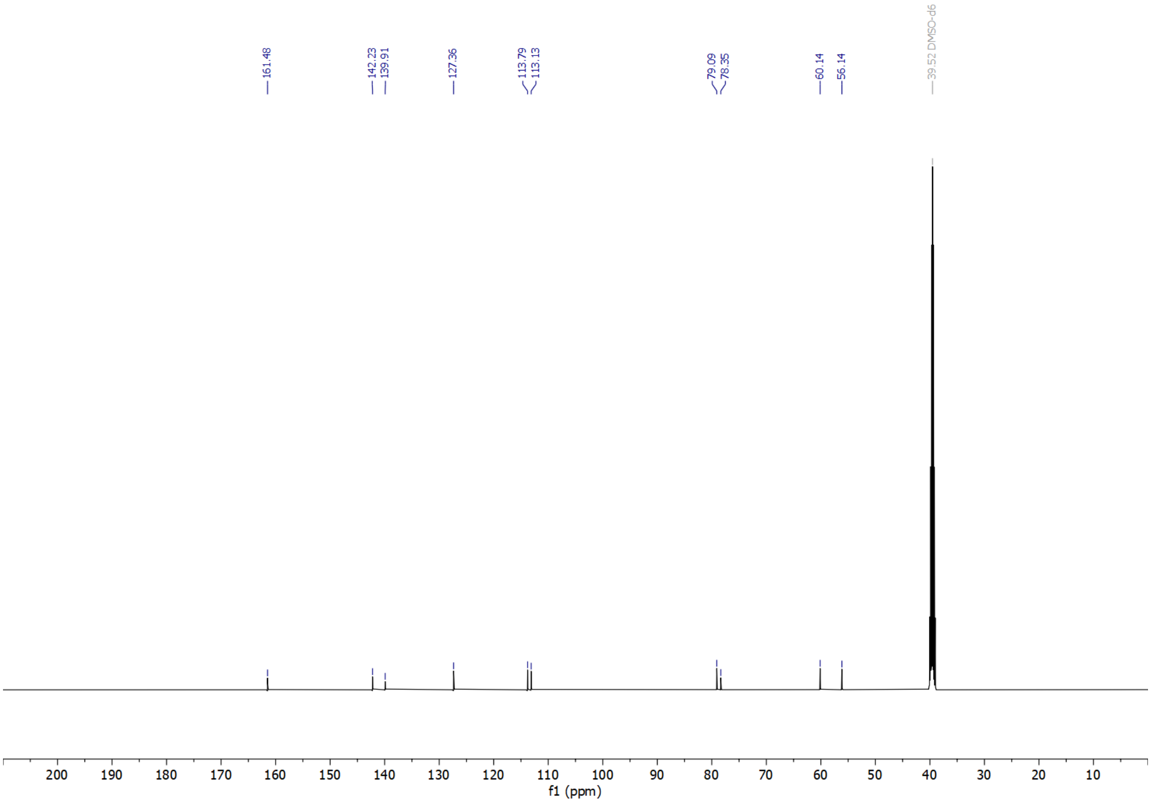

2,5-dioxo-1-pyrrolidinyl 4-(hydroxymethyl)-3-nitrobenzoate (*2*)

^{1}H NMR (500 MHz, DMSO-d_6)

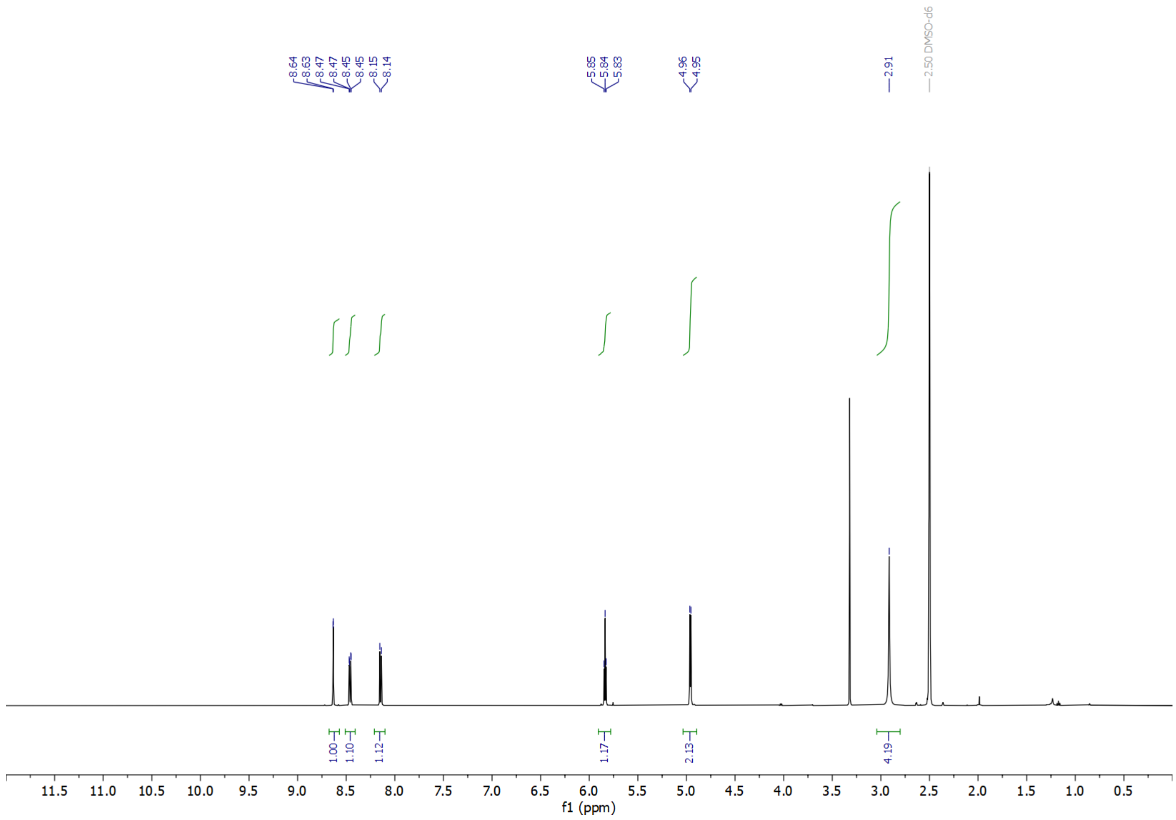

^{13}C NMR (101 MHz, DMSO-d_6)

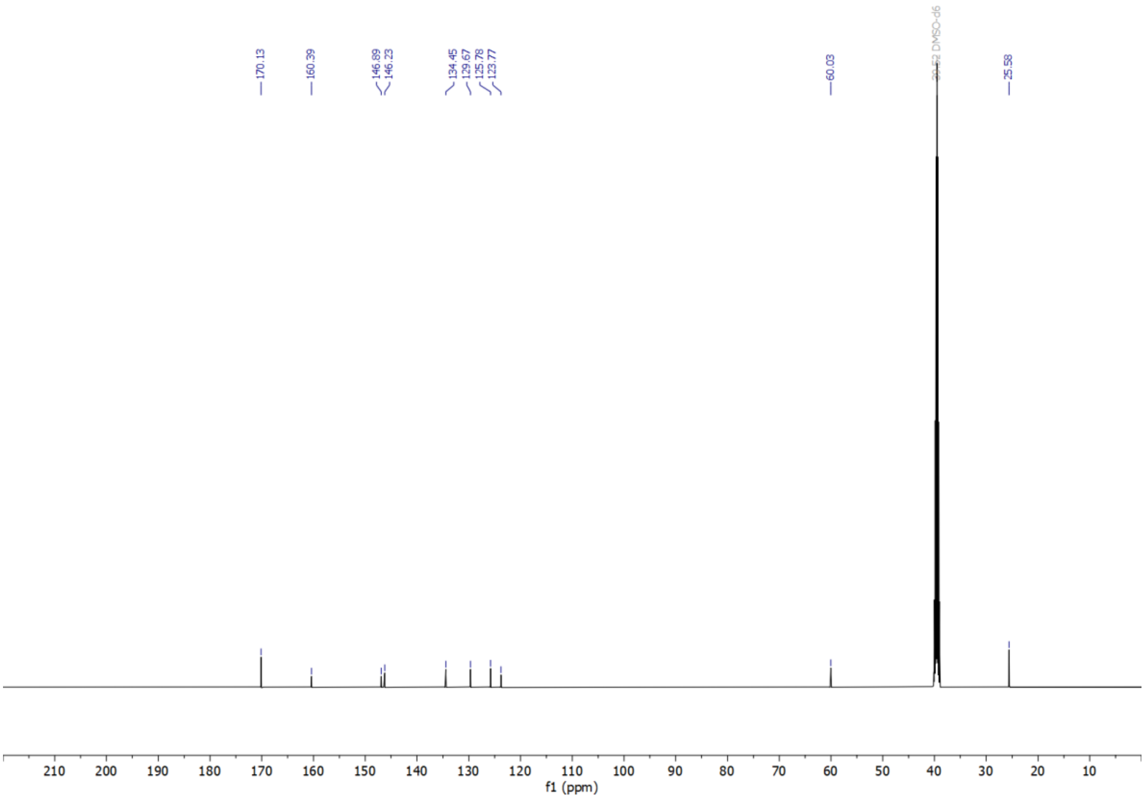

N,N'-1,2-ethanediylbis(4-hydroxymethyl-3-nitrobenzamide (*3*)

^{1}H NMR (500 MHz, DMSO-d_6)

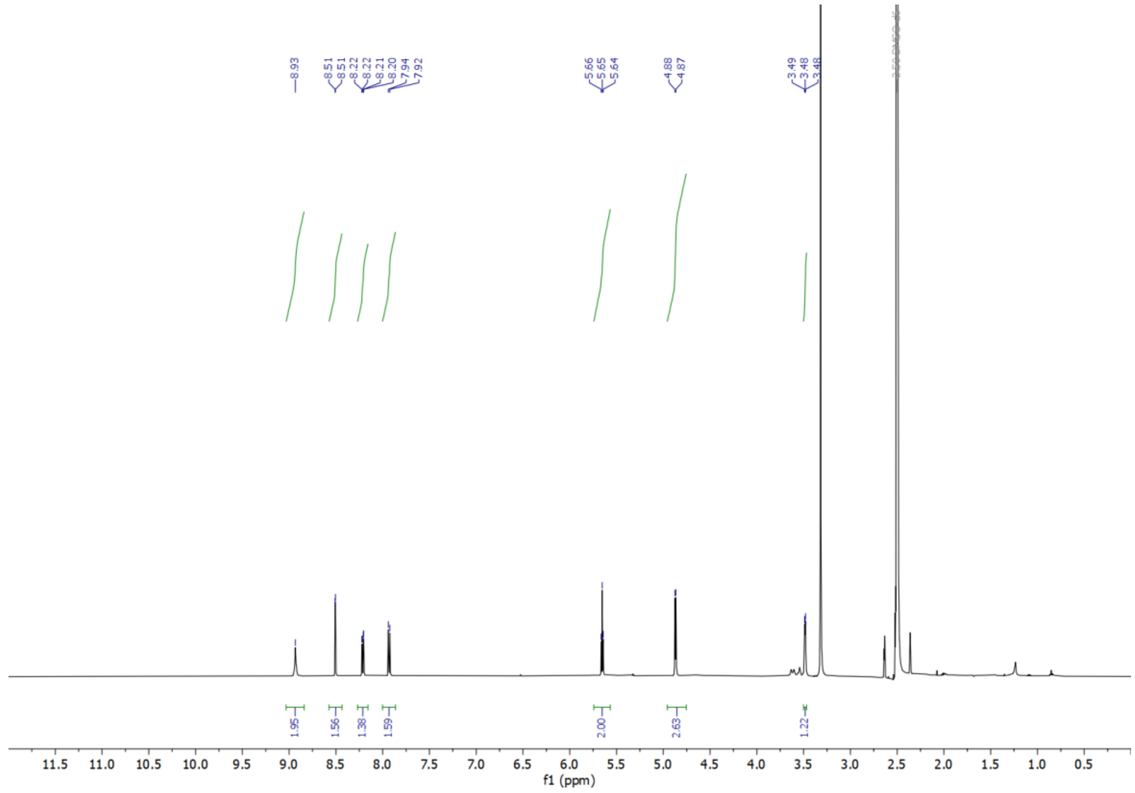

^{13}C NMR (101 MHz, DMSO-d_6)

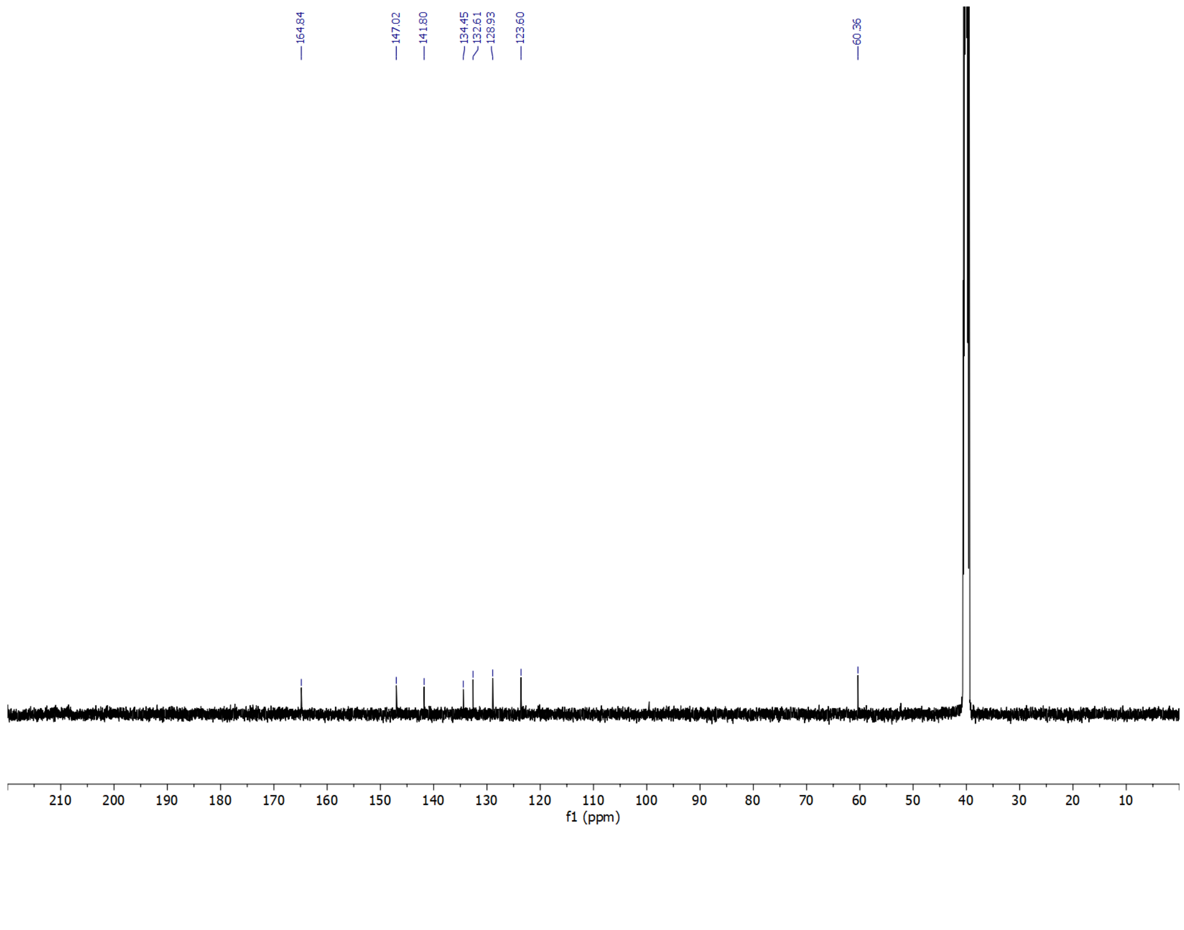

Supplementary Table 1. Yields of activation and reactivity of *o*NBA and intermediates determined via ^{1}H NMR. See Supplementary Figure 1 for reaction schematic which shows the chemical structure of the activated species B.

Entry	Time after irradiation (mins)	Additive	Time after additive addition (mins)	Yield of B (%)[a]
1	5	-	-	**46**
2	10	-	-	**47**
3	15	Lys.HCl[b]	5	**20**
4	20	Lys.HCl[b]	10	**7**
5	25	Lys.HCl[b]	15	**4**
6	5	-	-	**46**
7	10	-	-	**48**
8	15	Tris.HCl[b]	5	**32**
9	20	Tris.HCl[b]	10	**24**
10	25	Tris.HCl[b]	15	**26**
11	5	-	-	**47**
12	10	-	-	**46**
13	15	D_2O	5	**32**
14	20	D_2O	10	**27**
15	25	D_2O	15	**27**

[a]Determined by ^{1}H NMR spectroscopy with reference to mesitylene as an internal standard.
[b]7.5 equiv; 0.27M solution in D_2O.

Supplementary Table 2. Extent of labelling of SurA using *1b* determined from intact MS when the probe was added at different molar excesses. Data are shown as mean and standard deviation of three replicate measurements.

	10x		20x		30x		40x		50x	
**Modifications**	**Mean**	**SD**	**Mean**	**SD**	**Mean**	**SD**	**Mean**	**SD**	**Mean**	**SD**
**0**	71.377	3.929	51.558	9.415	46.880	1.877	32.062	2.220	26.450	8.440
**1**	21.181	2.017	27.313	1.380	33.645	1.589	33.489	1.054	31.583	1.598
**2**	6.080	1.331	12.939	1.012	14.283	2.353	20.362	0.738	21.165	2.068
**3**	0	0	4.564	4.127	4.655	4.072	9.081	1.109	12.194	1.177
**4+**	0	0	3.626	3.157	1.516	2.626	5.006	0.652	8.608	8.898

Supplementary Table 3. Extent of labelling of SurA using *1a* determined from intact MS when the probe was added at different molar excesses. Data are shown as mean and standard deviation of three replicate measurements.

	10x		20x		30x		40x		50x	
Modifications	Mean	SD	Mean	SD	Mean	SD	Mean	SD	Mean	SD
0	44.419	3.456	4.586	2.009	2.853	3.043	0	0	0	0
1	36.615	1.348	16.271	2.505	5.082	1.823	0	0	0	0
2	15.069	2.186	23.643	0.805	14.981	3.124	6.946	1.701	0	0
3	3.897	0.952	23.538	1.057	20.176	2.830	13.616	2.593	0	0
4+	0	0	31.962	4.448	56.908	10.535	79.439	3.494	100	0

Supplementary Table 4. Extent of labelling of SurA using *the* NHS-ester probe determined from intact MS when the probe was added at different molar excesses. Data are shown as mean and standard deviation of three replicate measurements.

	10x		20x		30x		40x		50x	
**Modifications**	**Mean**	**SD**	**Mean**	**SD**	**Mean**	**SD**	**Mean**	**SD**	**Mean**	**SD**
**0**	3.987	3.460	0	0	0	0	0	0	0	0
**1**	15.983	7.152	3.306	5.726	1.741	3.015	0	0	0	0
**2**	24.569	5.511	12.383	7.016	7.215	9.735	2.695	4.668	0	0
**3**	24.087	0.479	19.162	4.969	13.117	13.767	5.420	5.817	0	0
**4+**	31.374	15.702	65.149	17.605	77.927	26.472	91.885	10.479	100	0

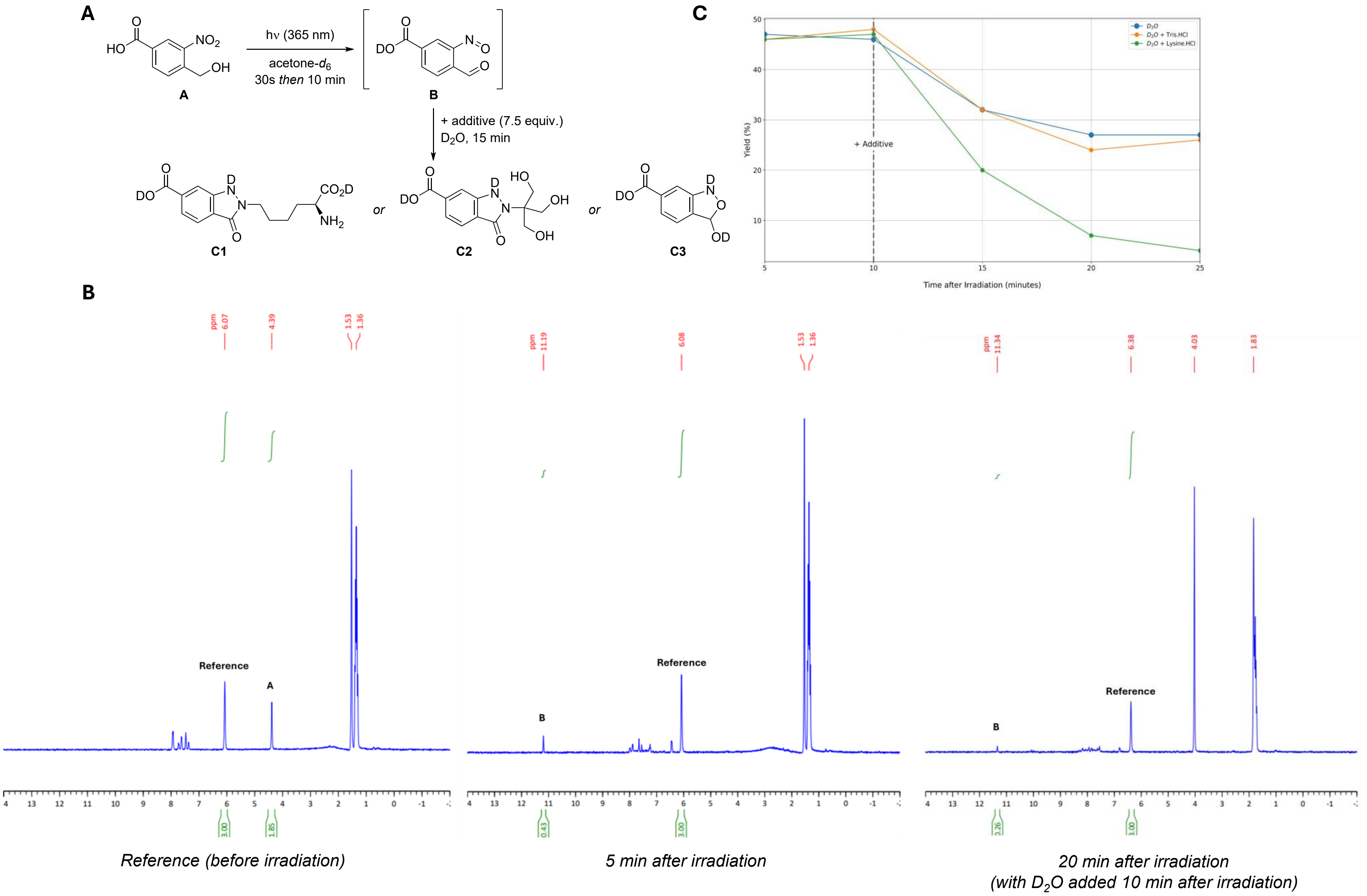

A
hν (365 nm)
acetone-d_6
30s *then* 10 min
A
B
+ additive (7.5 equiv.)
D_2O, 15 min
C1
or
C2
or
C3
C
+ Additive
Yield (%)
Time after Irradiation (minutes)
D_2O
D_2O + Tris.HCl
D_2O + Lysine.HCl
B
Reference
A
B
Reference
B
Reference
Reference (before irradiation)
5 min after irradiation
20 min after irradiation
(with D_2O added 10 min after irradiation)

Supplementary Figure 1. Yields of activation and reactivity of *o*NBA with D_2O, Tris.HCl and Lysine.HCl determined via ^1H NMR. (A) Reaction Schematic. After 30 seconds irradiation of 4-(Hydroxymethyl)-3-nitrobenzoic acid (A) at 365 nm, the resultant nitrosobenaldehyde (B) gives a diagnostic peak between 11.27 – 11.32 ppm. The quantification of this was against the internal standard of mesitylene highlighting the yield of the activated species and the rate of quenching by lysine, tris or water to form products C1, C2 and C3, respectively. **(B)** Exemplar ^1H NMR spectra of 4-(Hydroxymethyl)-3-nitrobenzoic acid before irradiation (left) and 5 min after irradiation (middle). The diagnostic peaks for the *o*NBA starting material (A) and the nitrosobenaldehyde (B) are annotated, as is the internal standard (reference). The spectra obtained 20 min after irradiation and with addition of D_2O is also shown (right). The intensity of peak B is reduced, consistent with quenching by water to form product C3 (see panel A). **(C)** Quantitation of yield of product B (i.e. the activated, reactive aldehyde) after irradiation (5 min timepoints). After 10 min, either D_2O, Tris.HCl or Lysine.HCl were added (see Methods), and the yield of the aldehyde was determined at 5 min intervals. Data used to generate this graph are shown in Supplementary Table 1.

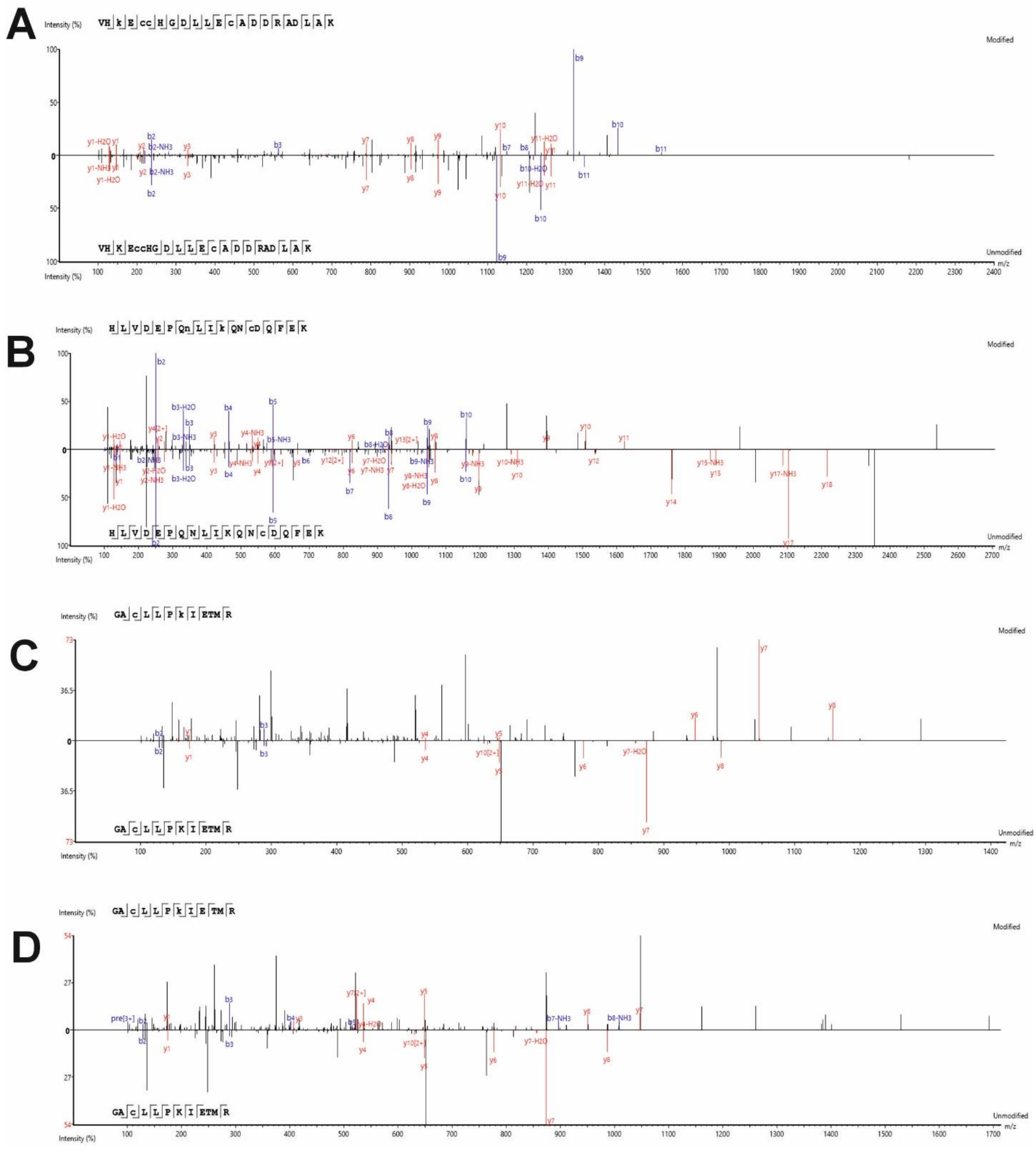

Supplementary Figure 2. Exemplar MS/MS spectra of modified and unmodified peptides from labelling BSA with probe *1a* and *1b*. (A) MS/MS spectrum of a tryptic peptide from BSA modified with the indazolone product using probe ***1a***. The MS/MS spectrum of the corresponding unmodified peptide is shown below. **(B)** MS/MS spectrum of a tryptic peptide from BSA modified with the secondary amine product using probe ***1a***. The MS/MS spectrum of the corresponding unmodified peptide is shown below. **(C)** MS/MS spectrum of a tryptic peptide from BSA modified with the indazolone product using probe ***1b***. The MS/MS spectrum of the corresponding unmodified peptide is shown below. **(D)** MS/MS spectrum of a tryptic peptide from BSA modified with the secondary amine product using probe ***1b***. The MS/MS spectrum of the corresponding unmodified peptide is shown below.

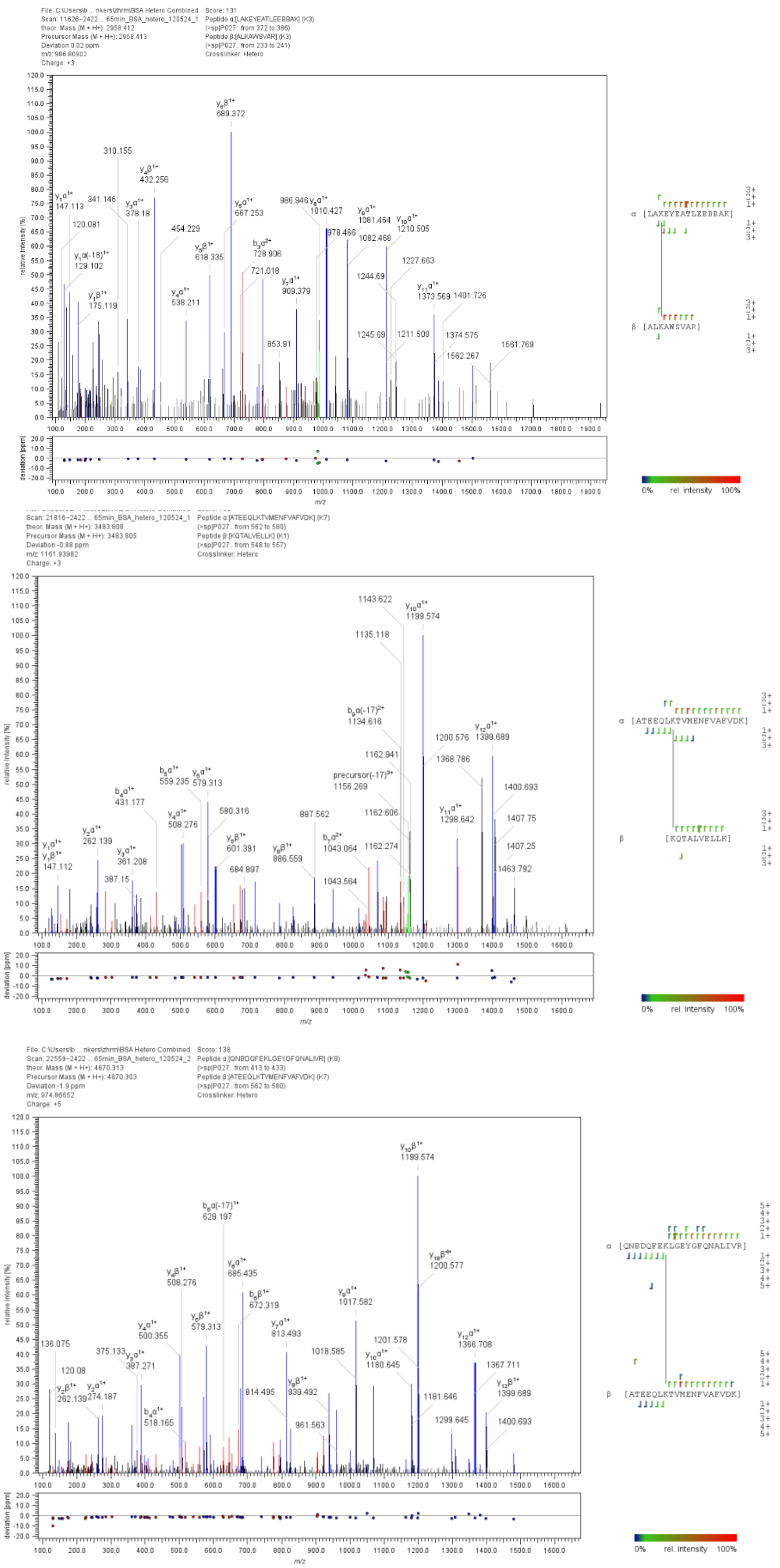

Supplementary Figure 3. Exemplar MS/MS spectra of identified crosslinks using crosslinker *2* with BSA.

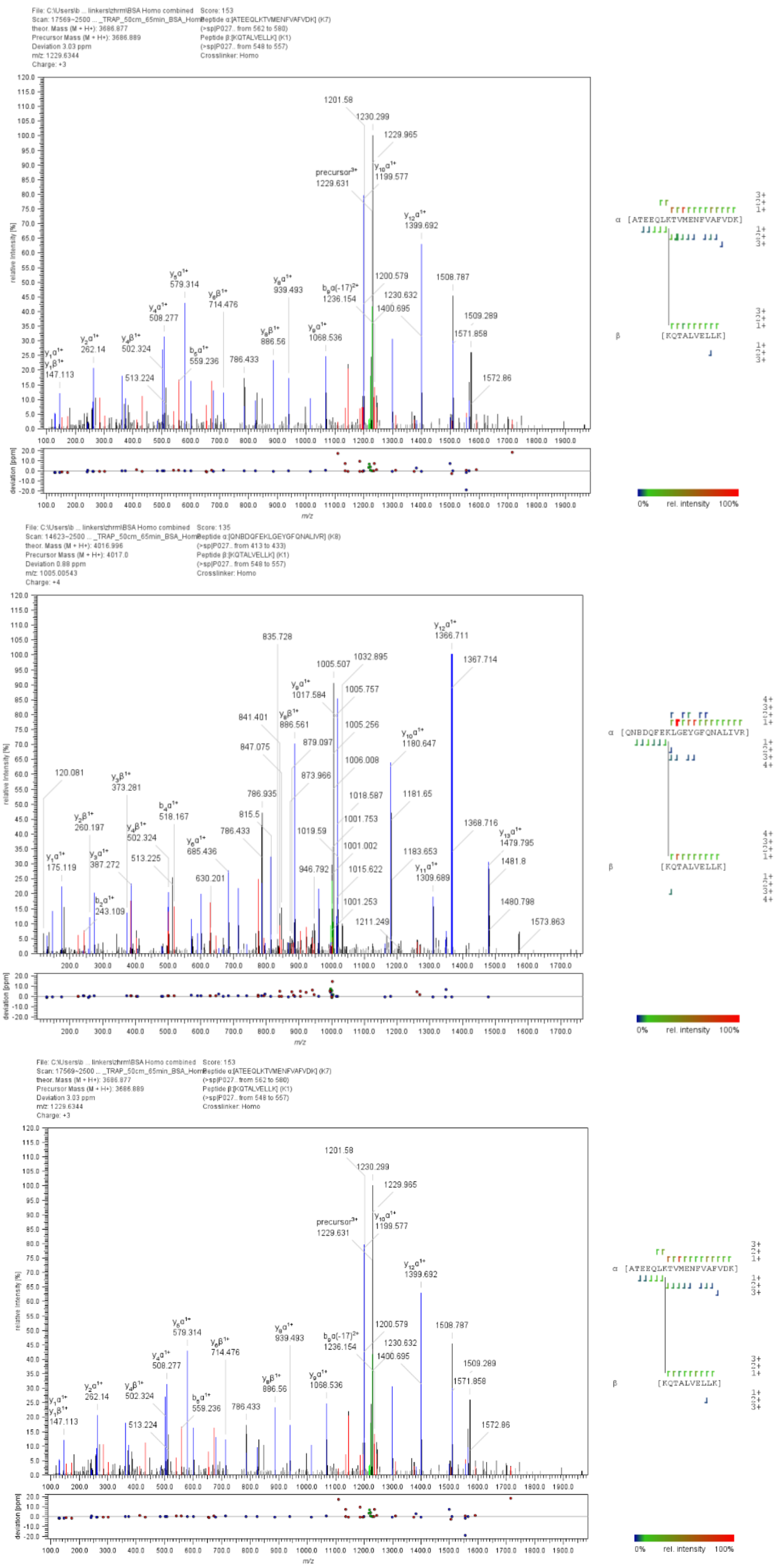

Supplementary Figure 4. Exemplar MS/MS spectra of identified crosslinks using crosslinker *3* with BSA.

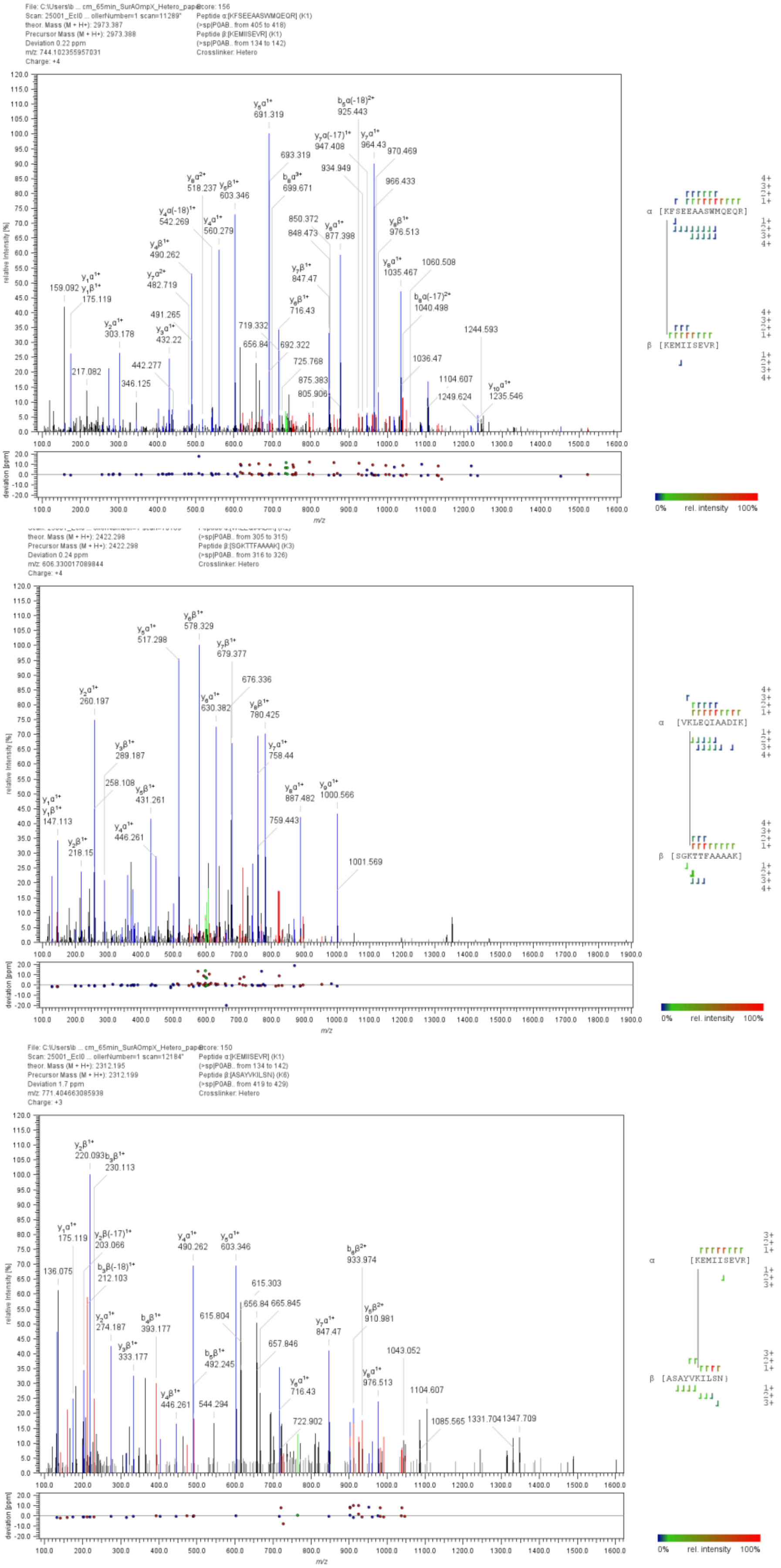

Supplementary Figure 5. Exemplar spectra of identified crosslinks using crosslinker *2* with SurA-OmpX.

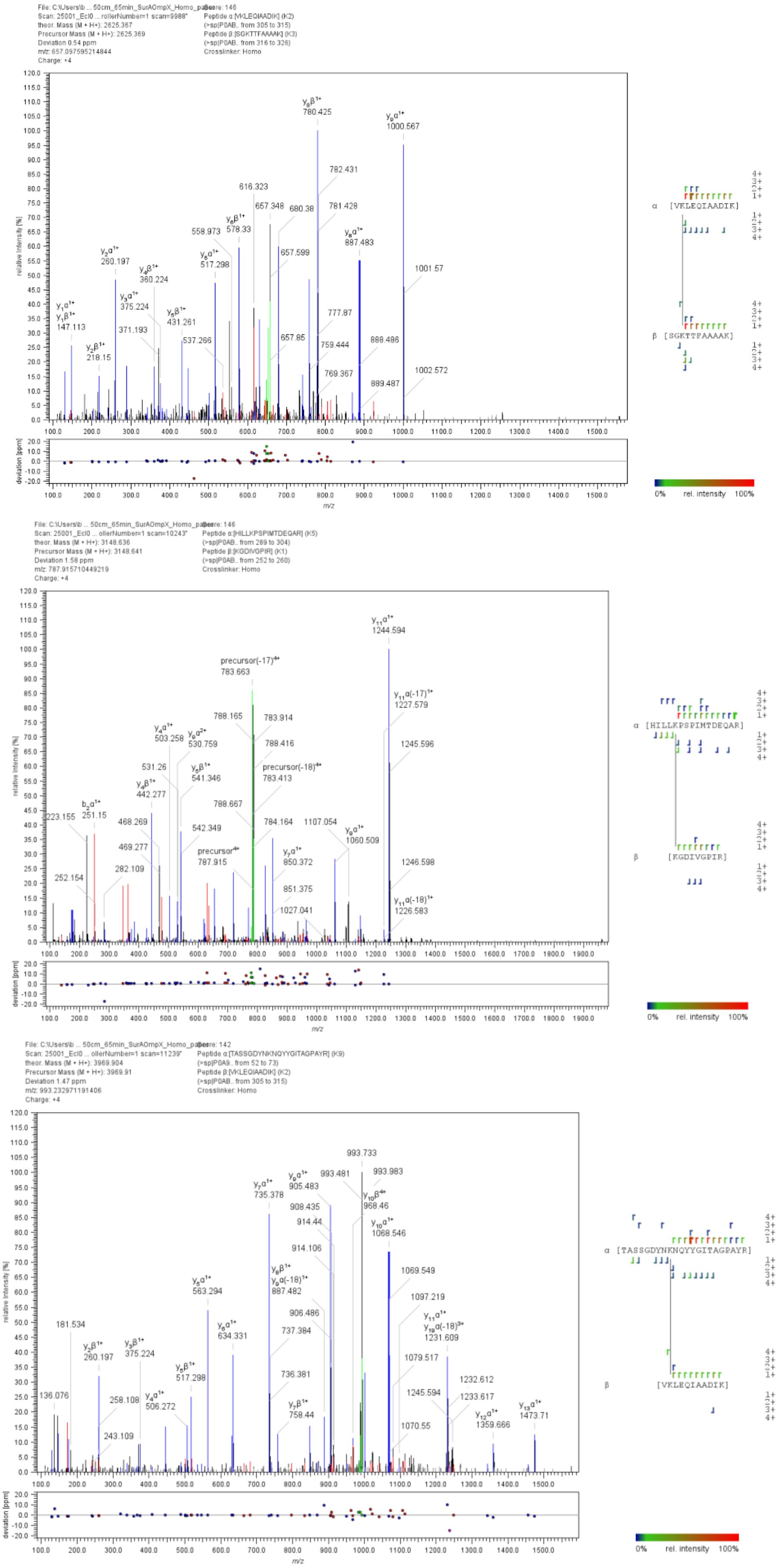

Supplementary Figure 6. Exemplar spectra of identified crosslinks using crosslinker *3* with SurA-OmpX.

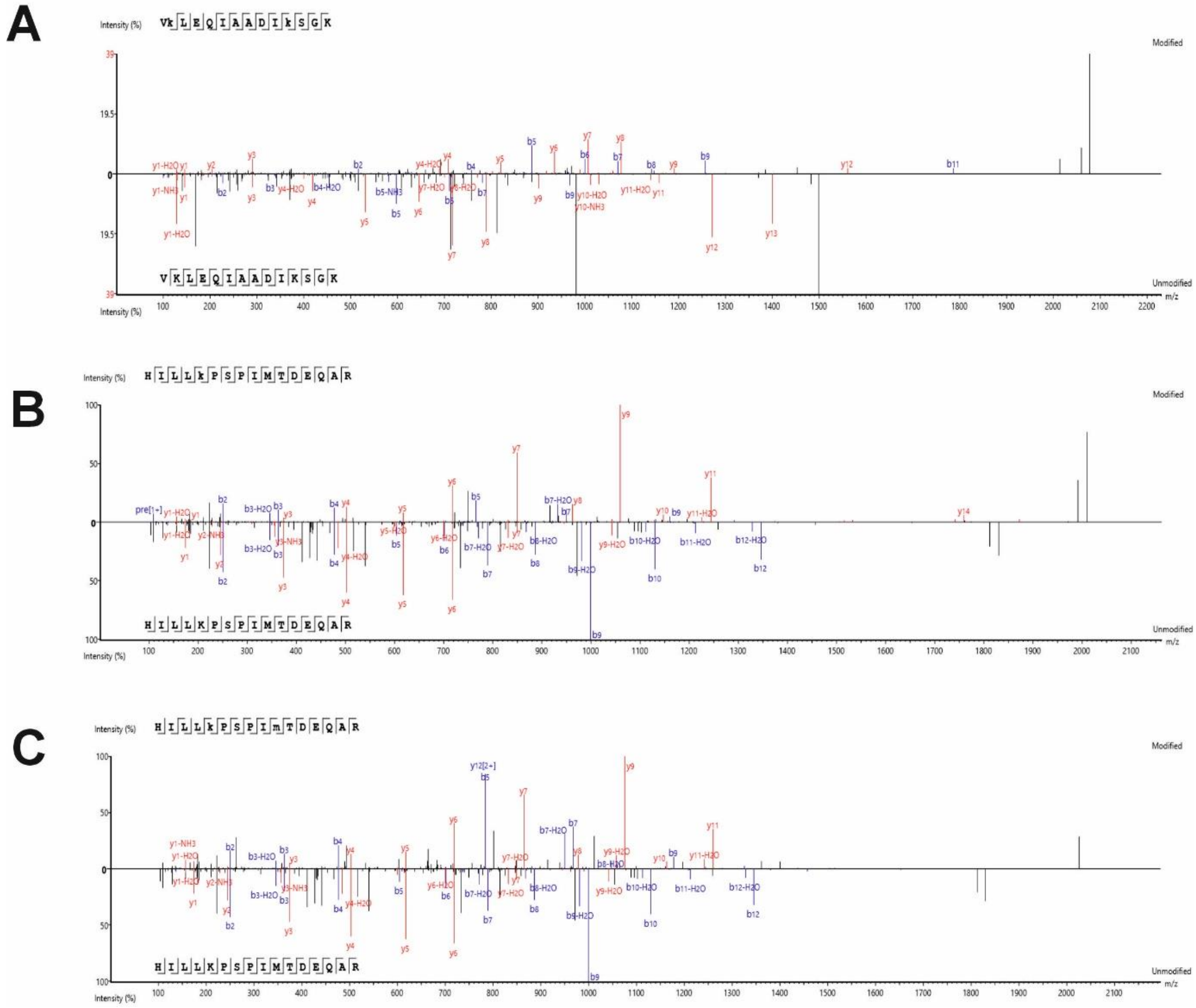

Supplementary Figure 7. Exemplar MS/MS spectra of mono-linked species and unmodified peptides from crosslinking SurA-OmpX using crosslinker *2*. (A) MS/MS spectrum of a tryptic peptide modified with the lysine quench product using crosslinker ***2***. The MS/MS spectrum of the corresponding unmodified peptide is shown below. **(B)** MS/MS spectrum of a tryptic peptide modified with crosslinker ***2*** (and no quenching). The MS/MS spectrum of the corresponding unmodified peptide is shown below. **(C)** MS/MS spectrum of a tryptic peptide modified with the hydrolysed quench product using crosslinker ***2***. The MS/MS spectrum of the corresponding unmodified peptide is shown below.

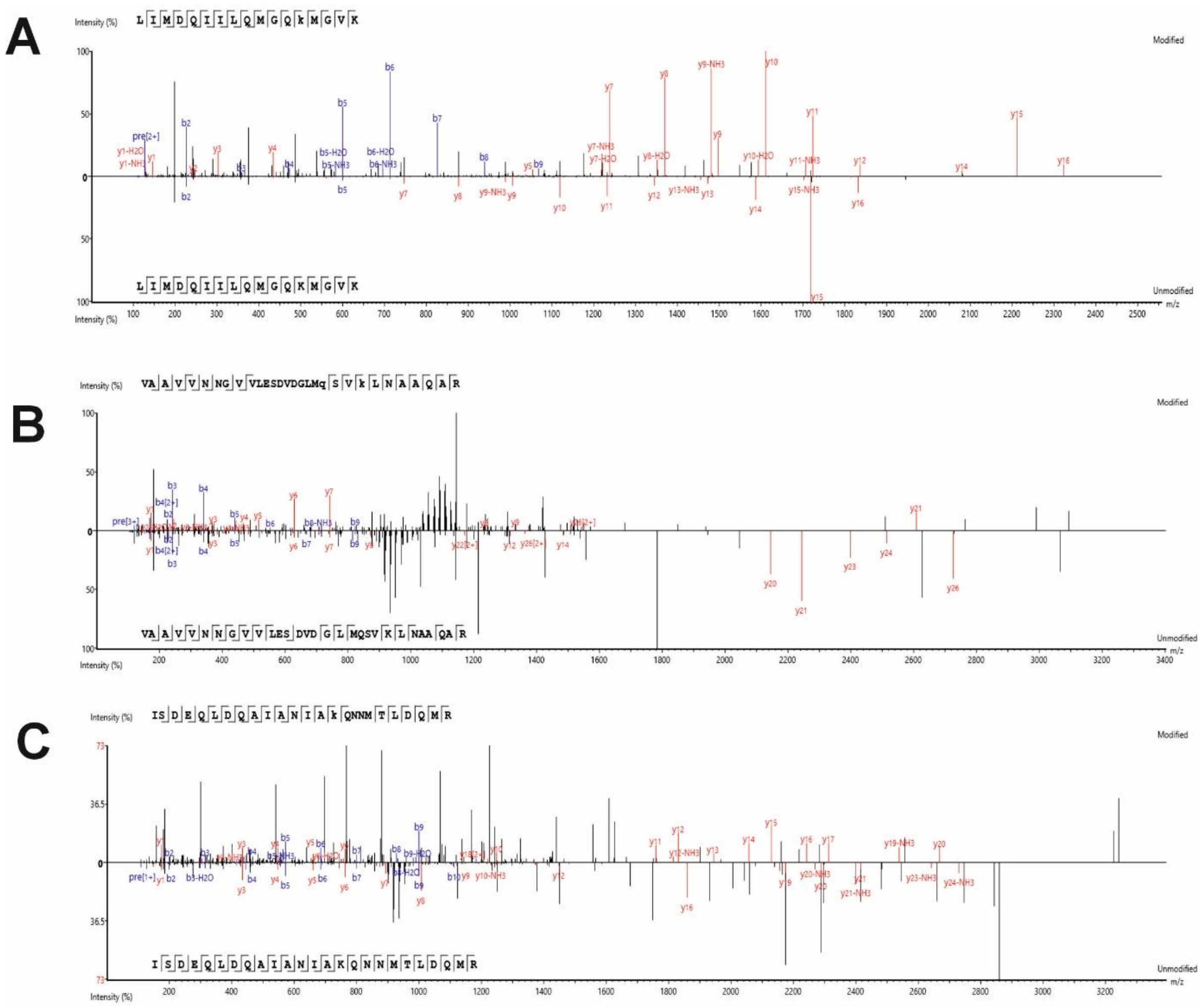

Supplementary Figure 8. Exemplar MS/MS spectra of mono-link species and unmodified peptide from crosslinking SurA-OmpX using crosslinker *3*. (A) MS/MS spectrum of a tryptic peptide modified with the lysine quench product using crosslinker ***3***. The MS/MS spectrum of the corresponding unmodified peptide is shown below. **(B)** MS/MS spectrum of a tryptic peptide modified with crosslinker ***3*** (and no quenching). The MS/MS spectrum of the corresponding unmodified peptide is shown below. **(C)** MS/MS spectrum of a tryptic peptide modified with the hydrolysed quench product using crosslinker ***3***. The MS/MS spectrum of the corresponding unmodified peptide is shown below.

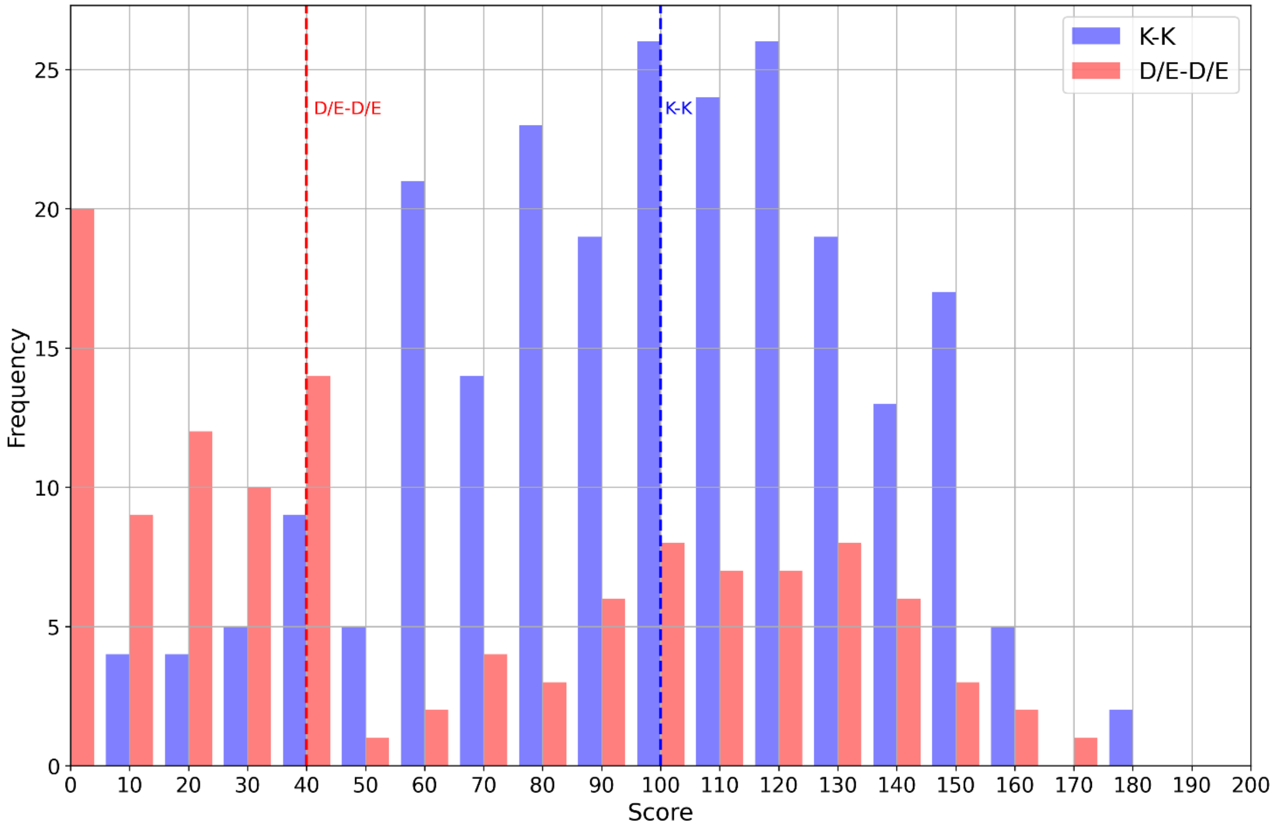

Supplementary Figure 9. Histogram of the MeroX scores of BSA crosslinks identified using *2*.

MS/MS spectra of crosslinks are given scores on their confidence of assignments based on sequence coverage, mass error and signal-to-noise. The score distributions represent CSMs when searching using either Lysine-Lysine specific parameters (blue) or parameters searching for crosslinks between acidic residues (red) from the MS/MS spectra of BSA using ***2***. Only lysine-lysine crosslinks can be present within the sample and the difference between the median scores (dotted lines) reflects the ability of ***2*** to produce confident assignments because the CSM scores are greater when searching for Lysine-Lysine crosslinks.

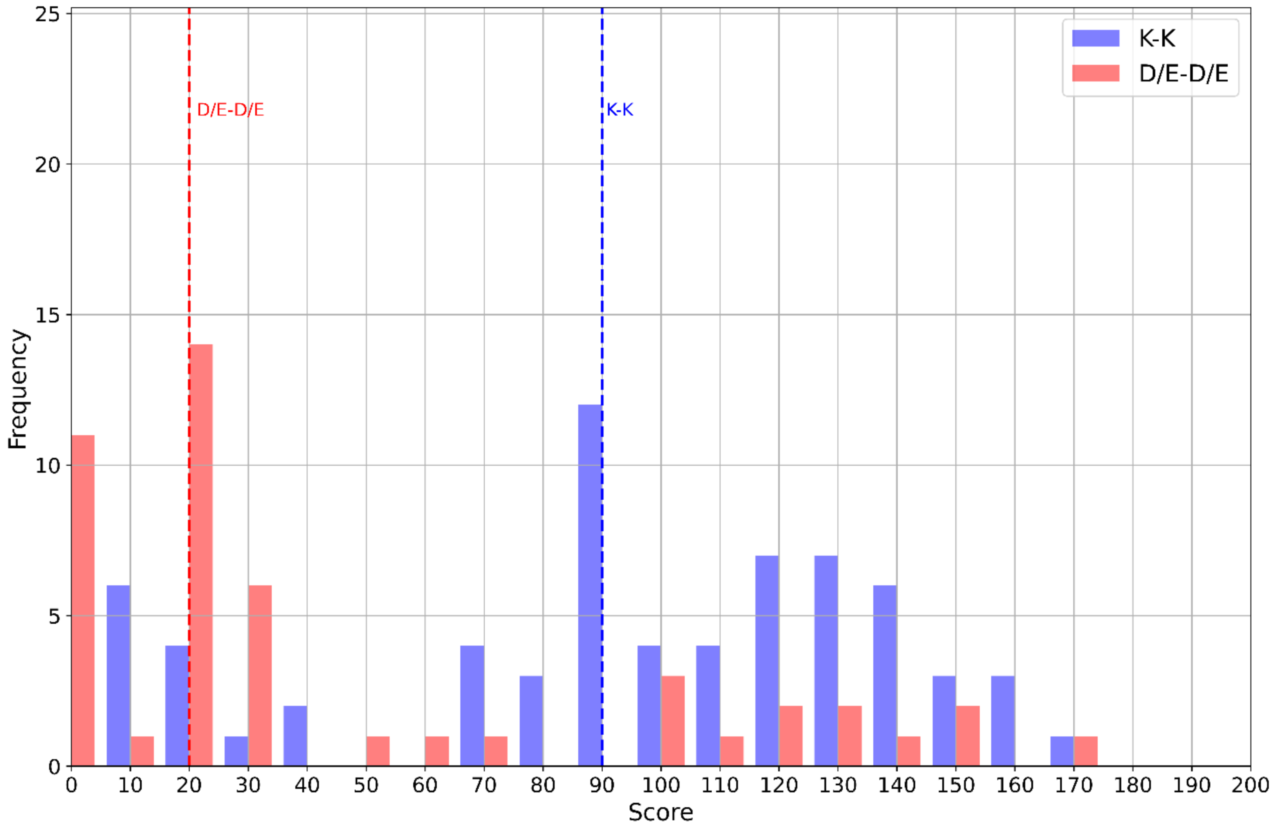

Supplementary Figure 10. Histogram of the MeroX scores of BSA crosslinks identified using *3*.

MS/MS spectra of crosslinks are given scores on their confidence of assignments based on sequence coverage, mass error and signal-to-noise. The score distributions represent CSMs when searching using either Lysine-Lysine specific parameters (blue) or parameters searching for crosslinks between acidic residues (red) from the MS/MS spectra of BSA using ***3***. Only lysine-lysine crosslinks can be present within the sample and the difference between the median score (dotted lines) reflects the ability of ***3*** to produce confident assignments because the CSM scores are greater when searching for Lysine-Lysine crosslinks.
